# The interaction between cannabis use and the Val158Met polymorphism of the COMT gene in psychosis: A transdiagnostic meta – analysis

**DOI:** 10.1371/journal.pone.0192658

**Published:** 2018-02-14

**Authors:** Thomas Stephanus Johannes Vaessen, Lea de Jong, Annika Theresia Schäfer, Thomas Damen, Aniek Uittenboogaard, Pauline Krolinski, Chinyere Vicky Nwosu, Florentina Maria Egidius Pinckaers, Iris Leah Marije Rotee, Antonius Petrus Wilhelmus Smeets, Ayşegül Ermiş, James L. Kennedy, Dorien H. Nieman, Arun Tiwari, Jim van Os, Marjan Drukker

**Affiliations:** 1 Student Faculty of Health Medicine and Life Sciences, Maastricht University, Maastricht, The Netherlands; 2 Department of Psychiatry, Bakirkoy Mazhar Osman Mental Health and Neurological Diseases Education and Research Hospital, Istanbul, Turkey; 3 Neurogenetics Section, Campbell Family Mental Health Research Institute, Centre for Addiction and Mental Health, Toronto, Ontario, Canada; 4 Department of Psychiatry, Academic Medical Center, Amsterdam, the Netherlands; 5 Department of Psychiatry and Psychology, School for Mental Health and Neuroscience (MHeNS), Maastricht University, Maastricht, The Netherlands; 6 Department Psychiatry, Brain Centre Rudolf Magnus, Utrecht University Medical Centre, Utrecht, The Netherlands; 7 King's College London, King's Health Partners Department of Psychosis Studies; Institute of Psychiatry, London, United Kingdom; Peking University, Institute of Mental Health, CHINA

## Abstract

**Background:**

Neither environmental nor genetic factors are sufficient to predict the transdiagnostic expression of psychosis. Therefore, analysis of gene-environment interactions may be productive.

**Objective:**

A meta-analysis was performed using papers investigating the interaction between cannabis use and catechol-O-methyl transferase (COMT) polymorphism Val158Met (COMT^Val158Met^).

**Data sources:**

PubMed, Embase, PsychInfo.

**Study eligibility criteria:**

All observational studies assessing the interaction between COMT^Val158Met^ and cannabis with any psychosis or psychotic symptoms measure as an outcome.

**Study appraisal and synthesis methods:**

A meta-analysis was performed using the Meta-analysis of Observational Studies in Epidemiology guidelines and forest plots were generated. Thirteen articles met the selection criteria: 7 clinical studies using a case-only design, 3 clinical studies with a dichotomous outcome, and 3 studies analysing a continuous outcome of psychotic symptoms below the threshold of psychotic disorder. The three study types were analysed separately. Validity of the included studies was assessed using "A Cochrane Risk of Bias Assessment Tool: for Non-Randomized Studies of Interventions".

**Results:**

For case-only studies, a significant interaction was found between cannabis use and COMT^Val158Met^, with an OR of 1.45 (95% Confidence Interval = 1.05–2.00; Met/Met as the risk genotype). However, there was no evidence for interaction in either the studies including dichotomous outcomes (B = -0.51, 95% Confidence Interval -1.72, 0.70) or the studies including continuous outcomes (B = -0.04 95% Confidence Interval -0.16–0.08).

**Limitation:**

A substantial part of the included studies used the case-only design, which has lower validity and tends to overestimate true effects.

**Conclusion:**

The interaction term between cannabis use and COMT^Val158Met^ was only statistically significant in the case-only studies, but not in studies using other clinical or non-clinical psychosis outcomes. Future additional high quality studies might change current perspectives, yet currently evidence for the interaction remains unconvincing.

## Introduction

Interaction between genes and environment may increase the risk to develop outcomes in the psychosis spectrum [1, 2]. Although the causes of the transdiagnostic expression of psychosis remain unknown [3], several risk factors have been identified. First, the transdiagnostic expression of psychosis, however defined, clusters in families [4, 5]. Second, environmental factors such as cannabis consumption also increase the risk of developing psychotic disorder or symptoms [6–8]. Genes and environment may reinforce each other’s effects; thus, it has been suggested that genetic variation may render an individual more sensitive to the psychotogenic effects of cannabis [9]. One example of gene-cannabis interaction is the hypothesized moderating effect of the catechol-O-methyl transferase (COMT) polymorphism Val158Met (COMT^Val158Met^) in the association between cannabis use and the emergence of the psychosis phenotype [10, 11]. The prevalence of cannabis consumption among patients with a diagnosis of psychosis is significantly higher than in the general population (42.1% lifetime use vs 22.5% lifetime misuse [12]). However, causal inference is difficult. For example, some patients use cannabis as a form of self-medication or to reduce the side effects of anti-psychotic medication [13]. On the other hand, causality may be plausible. The plant produces several compounds classified as cannabinoids, *Δ9-tetrahydrocannabinol* (THC) being the major psychoactive component. Animal studies have reported cannabis-associated alterations in dopaminergic neurotransmission both in the prefrontal cortex [14, 15] and in the mesolimbic pathway (reward system) [16].

Recent research suggests that GxE likely involves multiple genetic variants [17]. However, it has been advocated to perform replication studies analysing exactly the same hypothesis, phenotype and methodology to detect type I error [2]. Only the interaction between cannabis and COMT^Val158Met^ has been studied frequently enough to allow meta-analysis, although different study designs were employed. The COMT gene encodes for the enzyme catechol-O-methyl transferase (COMT), which is required for the catabolism of essential monoamines [18]. COMT^Val158Met^, also known as rs4680 is thought to alter synaptic availability of dopamine in the cortex, leading to memory and attention impairments and altered levels of dopamine signalling in the mesolimbic system, thus possibly moderating the risk of developing hallucinations and delusions [19, 20].

Given the fact that the same environmental and genetic effects appear to impact psychosis across different clinical and non-clinical levels of the psychosis spectrum [4, 21] we studied GxE in models of transdiagnostic expression of psychosis. In order to verify the validity of this approach, sensitivity analyses were conducted in groups of studies with comparable phenotypic outcomes.

When the primary interest of a study is to assess a possible interaction between genetic (COMT^Val158Met^) and environmental (cannabis) factors impinging on the development of psychosis, the use of case-only designs is one possibility. This method is used in several studies, such as the study by Costas and colleagues [22]. When a case-only study design to provide evidence for gene-environment interaction is used, the main assumption is that the prevalences of the environmental factor and the genotype are independent of each other in the population (no gene-environment correlation). The basic design is a simple 2x2 table as shown in Table 1, from which OR_co_ (odds ratio in Case-only design) can be calculated. To interpret the *OR_CO_* in the context of the case-only design, OR_CO_ is taken as a function of the OR of the exposure alone (OR_e_), the genotype alone (OR_g_), and the interaction effect OR (OR_g+e_) as would be examined in a case-control design. The formulae underlying the OR_CO_ is: OR_CO_ = OR_g+e_/((OR_e_ * OR_g_)* Z), where *Z* describes the odds ratio (OR) between exposure and genotype in the control group [23]. Since the main assumption of the case-only design is that exposure and genotype are independent of each other, *Z* = 1. At this point, the OR_CO_ obtained from the case-only design is no different than the synergy index obtained from a case-control design. Therefore, OR_CO_ describes departure from only multiplicative effects between the environmental exposure and genotype, similar to the regression coefficient for interaction in a case-control data set.

**Table 1:** Template for a case-only 2x2Table.

		Genotype susceptibility	
		+ (Met/Met)	- (Val/Val or Val/Met)
**Exposure to environmental factor (cannabis)**	yes	a	b
	no	c	d

OR = (a*d) / (b*c)

According to the continuum hypothesis, psychotic symptoms as an outcome of psychosis risk factors should be present not only in subjects diagnosed with psychotic disorder or schizophrenia, but also in subjects from the general population that do not fulfil the clinical criteria [24] and in people considered at ultra-high risk of psychosis [25, 26]. Ultra high risk populations are located on the continuum between the case-only and case-control studies on the one hand and the general population studies on the other. Attenuated psychotic experiences in an ultra high risk (UHR) sample often co-occur with common mental disorder or affective or anxiety symptoms [27]. Under this scenario, studying psychotic symptoms in the general population, in those at ultra-high risk and studying the full blown disorder would hypothetically provide the same evidence for risk factors. This is the reason that in the search the definition of the outcome was broad, including the transdiagnostic expression of psychosis.

### Aim

The hypothesis was that evidence from gene-environment interaction between cannabis use and the COMT^Val158Met^ polymorphism would be apparent in studies of the transdiagnostic expression of psychosis. The present meta-analysis aimed to provide an overview of the current literature on this GxE interaction, as well as to provide pooled measures of this interaction. The primary measure of the interaction term between cannabis use and COMT^Val158Met^ impacting the development of psychosis was analysed and evaluated. Because the available studies used three different types of designs and associated phenotypic measures, generalised measures were calculated for each type of study separately: case-only, dichotomous clinical outcomes and continuous outcomes. Only studies using a white ethnic group were included in the present meta-analysis, because the moderating effects of COMT^Val158Met^ likely differ between different ethnic groups [28].

## Methods

### Data sources

The meta-analysis was conducted according to the Meta-analysis of Observational Studies in Epidemiology (MOOSE) guidelines [29]. In order to identify all suitable studies, the databases PubMed, Psychinfo and Embase were searched up to July 18^th^ of 2017. Various combinations of the main keywords specifically “psychotic disorders”, “schizophrenia”, “psychosis”, “psychotic”, “catechol-o-methyltransferase”, “COMT”, “Val158Met” and “cannabis” were used. The Medical Subject Heading (MeSH) terms further helped with the specification of the search results. Subsequently, the ‘OR’ term was used to combine synonyms in order to yield more results. Second, the ‘AND’ term was used to obtain hits that include at least one term of all three categories (psychosis phenotype, COMT, cannabis). Table 2 illustrates the results of the PubMed search, in particular the search terms used on the left as well as the number of articles found on the right side.

**Table 2:** PubMed search results (July 18, 2017).

Search	Add to builder	Query	Items found
#33	Add	Search **(((((((("Schizophrenia"[Mesh]) OR ((Etiology/Broad[filter]) AND ("Schizophrenia"[Mesh]))) OR "Psychotic Disorders"[Mesh]) OR Schizophrenia) OR psychosis) OR psychotic)) AND ((((("Catechol O-Methyltransferase"[Mesh]) OR Catechol-O-Methyltransferase) OR COMT) OR "COMT protein, human" [Supplementary Concept]) OR Val158Met)) AND (((cannabis) OR marijuana) OR tetrahydrocannabinol)**	41
#32	Add	Search **((((((("Schizophrenia"[Mesh]) OR ((Etiology/Broad[filter]) AND ("Schizophrenia"[Mesh]))) OR "Psychotic Disorders"[Mesh]) OR Schizophrenia) OR psychosis) OR psychotic)) AND ((((("Catechol O-Methyltransferase"[Mesh]) OR Catechol-O-Methyltransferase) OR COMT) OR "COMT protein, human" [Supplementary Concept]) OR Val158Met)**	852
#31	Add	Search **((cannabis) OR marijuana) OR tetrahydrocannabinol**	29535
#30	Add	Search **(((("Catechol O-Methyltransferase"[Mesh]) OR Catechol-O-Methyltransferase) OR COMT) OR "COMT protein, human" [Supplementary Concept]) OR Val158Met**	6348
#29	Add	Search **((((("Schizophrenia"[Mesh]) OR ((Etiology/Broad[filter]) AND ("Schizophrenia"[Mesh]))) OR "Psychotic Disorders"[Mesh]) OR Schizophrenia) OR psychosis) OR psychotic**	178274
#28	Add	Search **tetrahydrocannabinol**	8086
#27	Add	Search **marijuana**	25659
#26	Add	Search **cannabis**	16638
#25	Add	Search **Val158Met**	829
#24	Add	Search **"COMT protein, human" [Supplementary Concept]**	328
#21	Add	Search **COMT**	4460
#20	Add	Search **Catechol-O-Methyltransferase**	5266
#18	Add	Search **"Catechol O-Methyltransferase"[Mesh]**	3725
#15	Add	Search **psychotic**	60086
#11	Add	Search **psychosis**	72835
#10	Add	Search **Schizophrenia**	127242
#9	Add	Search **"Psychotic Disorders"[Mesh]**	46545
#6	Add	Search **(Etiology/Broad[filter]) AND ("Schizophrenia"[Mesh])**	34928
#3	Add	Search **"Schizophrenia"[Mesh]**	94612

### Inclusion and exclusion criteria

All publication years in the databases and articles in English, German and Dutch were included in the search. In addition, no exclusion was conducted based on the type of study. It was decided beforehand that studies would be excluded in case of insufficient data. When information was not available (and was not provided after an e-mail and two reminders to the authors) the study was excluded. In order to ensure methodological quality of the studies, the risk of bias was assessed by two independent reviewers using the ACROBAT-NRSI (A Cochrane Risk of Bias Assessment Tool: for Non-Randomized Studies of Interventions) [30]. Socioeconomic status, age and sex were *a priori* set as important confounding factors and childhood trauma was identified as a co-exposure.

### Data extraction

Selected studies were stratified based on their type of outcome: case-only, dichotomous outcome or continuous outcome, given that pooling of odds ratios and linear regression coefficients in a single meta-analysis is complex. Data on the cannabis-COMT^Val158Met^ interaction were extracted (each study by 2 authors, independently).

Cannabis was a dichotomous variable in all studies. The present study defined Met/Met as the risk genotype (Met/Met = 1; Val/Val and Val/Met = 0), following the majority of the studies. Results of the case-only study defining Val/Val as the risk genotype [31] were recoded to obtain a 2x2 table similar to the other studies; conservatively ignoring the possibility of flip-flop [32]. As opposed to the case-only, all studies analysing dichotomous and continuous outcomes analysed COMT^Val158Met^ coded 0, 1, 2. For the present analysis, all results were recoded so that 0 was Val/Val, 1 was Val/Met and 2 was Met/Met. COMT^Val158Met^ was analysed as a continuous variable, suggesting equal effect sizes when comparing Val/Met to Val/Val and Met/Met to Val/Met.

For case-only studies, the odds ratios of the 2x2 cannabis by COMT^Val158Met^ table (Table 1) were extracted as a primary measure of the GxE interaction. When the authors did not provide the odds ratio, it was calculated from the 2x2 crosstabs. For the dichotomous outcomes, the coefficient (obtained from logistic regression) of the Cannabis X COMT^Val158Met^ interaction and its standard error were extracted (i.e. the exponent of the odds ratio usually reported after logistic regression). For the continuous outcomes, linear regression coefficients of the Cannabis X COMT^Val158Met^ interaction term and its standard error were extracted.

In addition, variables that could potentially modify the results were extracted, including diagnosis of patients (e.g. DSM IV), assessment of cannabis exposure and sex. Hypothetically, those variables can modify the association as defined in the research question.

### Statistical analysis

All analyses were performed using Stata version 13 [33]. The Stata command *metan* generated forest plots for each group of studies using random effects (DerSimonianLaird method).

The *metan* command also provided the between study variance (tau-square) and the Higgins I-square which is a measure of heterogeneity. When sufficient data was available (case-only studies), modifiers were analysed using the *metareg* command (diagnosis using DSM IV or revised DSM IV, cannabis use never versus ever or less stringent criteria, >70% male sex vs <70% male sex). The *metainf* command was used to check single study effects. Finally, publication bias was tested using the *metafunnel* command to generate a funnel plot, the *metatrim* command to identify the possibility of unpublished negative findings and to control for that (trim and fill) [34] as well as the *metabias* command to obtain Egger's test for small study effects.

Because only three studies analysing continuous outcomes were included (4 samples), a meta-regression model including the modifying effect of the variable study (using 2 dummies) was analysed rather than the three modifiers, separately. Study was not a modifier (F = 5.20, df = 2,1, p = 0.30), but visual inspection showed that results from the ultra-high risk population [35] were different.

### Sensitivity analyses

The present meta-analysis aimed to study the full psychosis spectrum, i.e. at the level of full blown disorder as well as at the level of psychotic symptoms below the clinical threshold. When studies with samples at different levels of the psychosis spectrum were included in the same meta-analysis, sensitivity analyses were performed including the majority of studies assessing the same population type. For example, the study analysing an ultra-high risk population was excluded in the continuous outcomes sensitivity analysis, the studies with phenotypes at the level of psychotic experiences in the general population remaining in the analysis.

## Results

**Fig 1:**
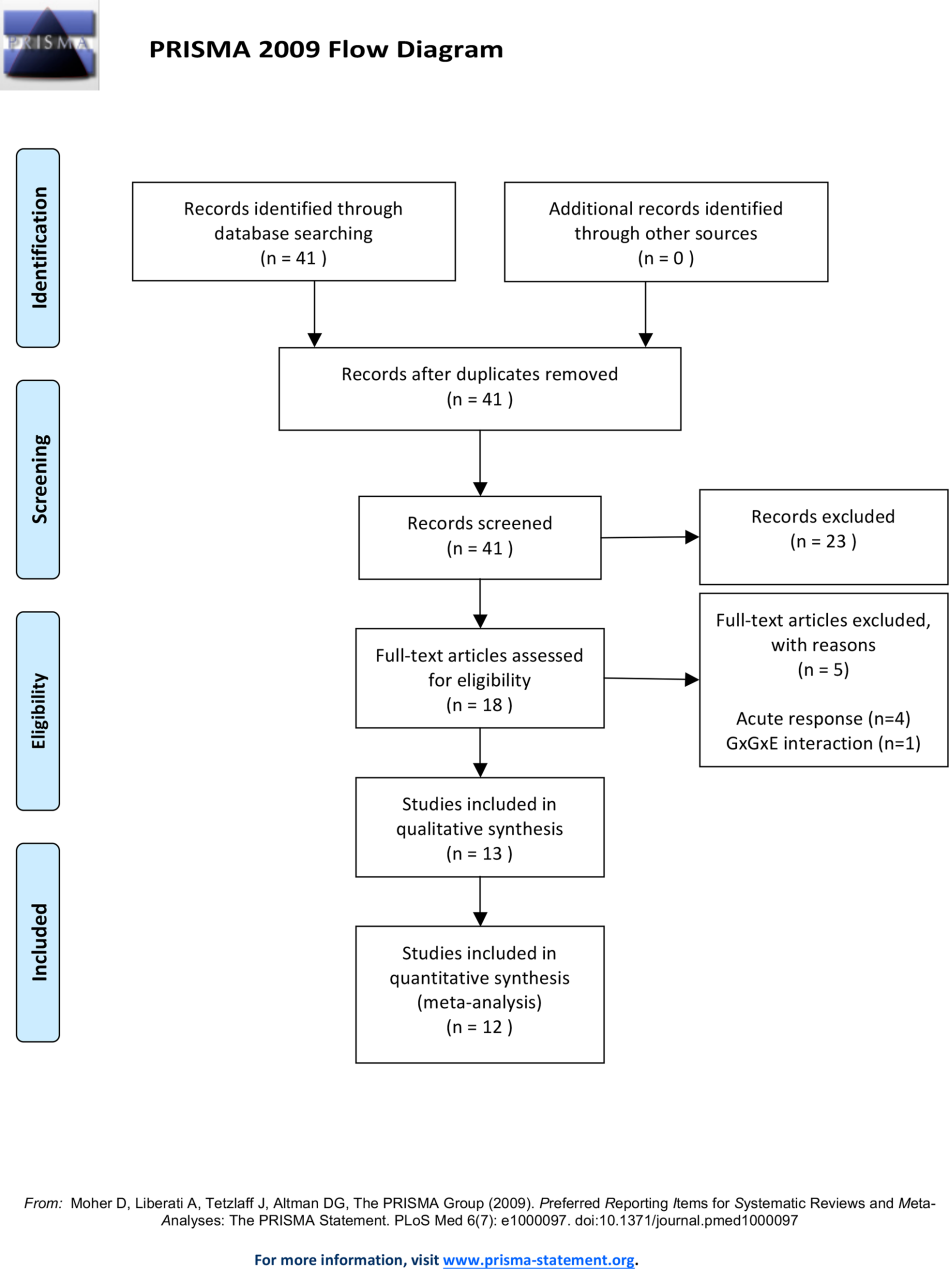
Flow diagram.

A total of 41 articles were selected based on the inclusion of the three search terms (Table 2, Fig 1). After searching PubMed, searching Psychinfo and Embase didn't provide any extra studies. After the initial search, backward citation tracking was utilised in order to ensure that all relevant studies were identified. However, no further research articles were found. After applying the aforementioned exclusion criteria, 13 articles were included in the final selection (7 case-only studies [22, 28, 31, 36–39], 2 studies analysing dichotomous outcomes [10, 40] and 3 analysing continuous outcomes [35, 41, 42]). A third study analysing dichotomous outcomes [43] was excluded from the meta-analysis because the data needed for extraction were not provided and the authors did not respond to requests for additional data. Authors of all articles checked for Hardy Weinberg equilibrium in order to ensure that the genotyping was done correctly.

### Case-only studies

Table 3 presents background information of the 7 case-only studies (n = 1954). One study presented results using two different populations, which are both included in the meta-analysis [22].

**Table 3:** Descriptive statistics case-only.

Article	Sample size	Assessment cannabis use	Assessment patients	Original coding of COMT^Val158Met^	Ethnicity	Sex (% male)
Costas 2011 [22]	Santiago: 382 Valencia: 365	Santiago: Lifetime prevalence of cannabis abuse according to DSM IV criteria determined by psychiatrist (blinded); Valencia: Cannabis use as assessed by medical records and confirmed by senior clinical psychologist	All patients meet DSM IV criteria for schizophrenia as determined by experiences psychiatrists	original paper: Met/Met, Val/Met, Val/Val, for analysis recoded into 1 Met/Met 0 Val/Val and Val/Met	South-western European	Santiago: 63% Valencia: 66%
De Sousa 2013 [37]	351	Never/less than once a month/weekly/more than weekly/daily in analyses never vs ever	DSM IV of schizophrenia or schizo-affective disorder-depressive type (using the Structured Clinical Interview for DSM-IV SCID-I/P)	original paper: Val/Val, Val/Met, Met/Met, for analysis recoded into 1 Met/Met 0 Val/Val and Val/Met^1^	European ancestry, Caucasian	72%
Ermis 2015 [39]	74	At least 5 times or more	DSM IV TR schizophrenia	original paper: Val/Val, Val/Met, Met/Met, for analysis recoded into 1 Met/Met 0 Val/Val and Val/Met^1^	Turkish	100%
Estrada 2011 [38]	80	Lifetime cannabis use: cannabis use (daily, weekly, monthly) or non-cannabis use (never or experimental consumption)	80 patients with schizophrenia-spectrum disorders, DSM-IV-TR	original paper and extra data^1^: Val/Val, Val/Met, Met/Met, for analysis recoded into 1 Met/Met 0 Val/Val and Val/Met	Caucasian	61%
Kantrowitz 2009 [28]	38	Adolescent cannabis use: defined as any use more than once prior to age 18	Caucasians and African-Americans: Structured Clinical Interview (SCID) for DSM-IV Axis I diagnosis of schizophrenia, schizoaffective disorder or psychosis	original paper: Met/Met, Val/Met, Val/Val, for analysis recoded into 1 Met/Met, 0 Val/Val and Val/Met	Only Caucasians used	87%
Pelayo-Teran 2010 [31]	169	Those who had been consuming 1 or more units (1 joint) per week in the previous year before the inclusion of study	First episode psychosis patients (meeting DSM–IV criteria for brief psychotic disorder, schizophreniform disorder, schizophrenia or schizoaffective disorder)	Val/Val, Val/Met, Met/Met for analysis recoded into 1 Met/Met, 0 Val/Val and Val/Met	European ancestry, Caucasian	59%
Zammit 2007 [36]	493	At least once	Diagnosis of schizophrenia according to DSM-IV	Additive model Met/Met, Val/Met, Val/Val. In analysis 1 Met/Met, 0 Val/Val and Val/Met^1^	White (both parents born Ireland/UK)	No information

^1^ After obtaining additional data from the authors

Validity of the case-only studies is presented in Table 4. As explained earlier, the design of a case-only study does not include a control group. The difficulty to find a good control group is one reason for the existence of case-only studies (20). Distribution of confounders in the non-existing control group is per definition the same in the patients. In addition, the question whether controls were sampled from the same population as the patients is not applicable (selection of participants). Three studies [36, 37, 39] did not describe how cannabis use was assessed and, therefore, scored “moderate” on “measurement of intervention” (i.e. the exposures cannabis and COMT^Val158Met^ as defined in the NRSI criteria list [30]). None of the studies used statistical methods to account for missing data, whilst most had incomplete data. A more detailed appraisal of the validity is available upon request.

**Table 4:** Validity assessment using the ACROBAT-NRSI [30].

	Confounding	Selection of participants	Measurement of interventions	Departures from intended interventions	Missing data	Measurement of outcomes	Selection of results
***Case-only***							
Costas 2011	low	n/a	Low	Low	Low	Low	Low
De Sousa 2013	low	n/a	Moderate	Low	Moderate	Low	Low
Ermis 2015	low	n/a	Moderate	Low	Low	Low	Low
Estrada 2011	low	n/a	Low	Low	Moderate	Low	Low
Kantrowitz 2009	low	n/a	Low	Low	Moderate	Low	Low
Pelayo-Teran 2010	low	n/a	Low	Low	Moderate	Low	Low
Zammit 2007	low	n/a	Moderate	Low	Moderate	Low	Low
***Dichotomous outcome***							
Caspi 2005	Moderate	Low	Moderate	Low	Moderate	Low	Low
Gutiérrez 2009	Moderate	Moderate	Moderate	Low	Moderate	Low	Low
Zammit 2011	Low	Low	Low	Low	Moderate	Moderate	Low
***Continuous outcome***							
Alemany 2014	Moderate	Low	Moderate	Low	Moderate	Moderate	Low
Nieman 2016	Moderate	Low	Moderate	Low	Moderate	Moderate	Low
Vinkers 2013	Moderate	Low	Moderate	Low	Low	Moderate	Low

**Fig 2:**
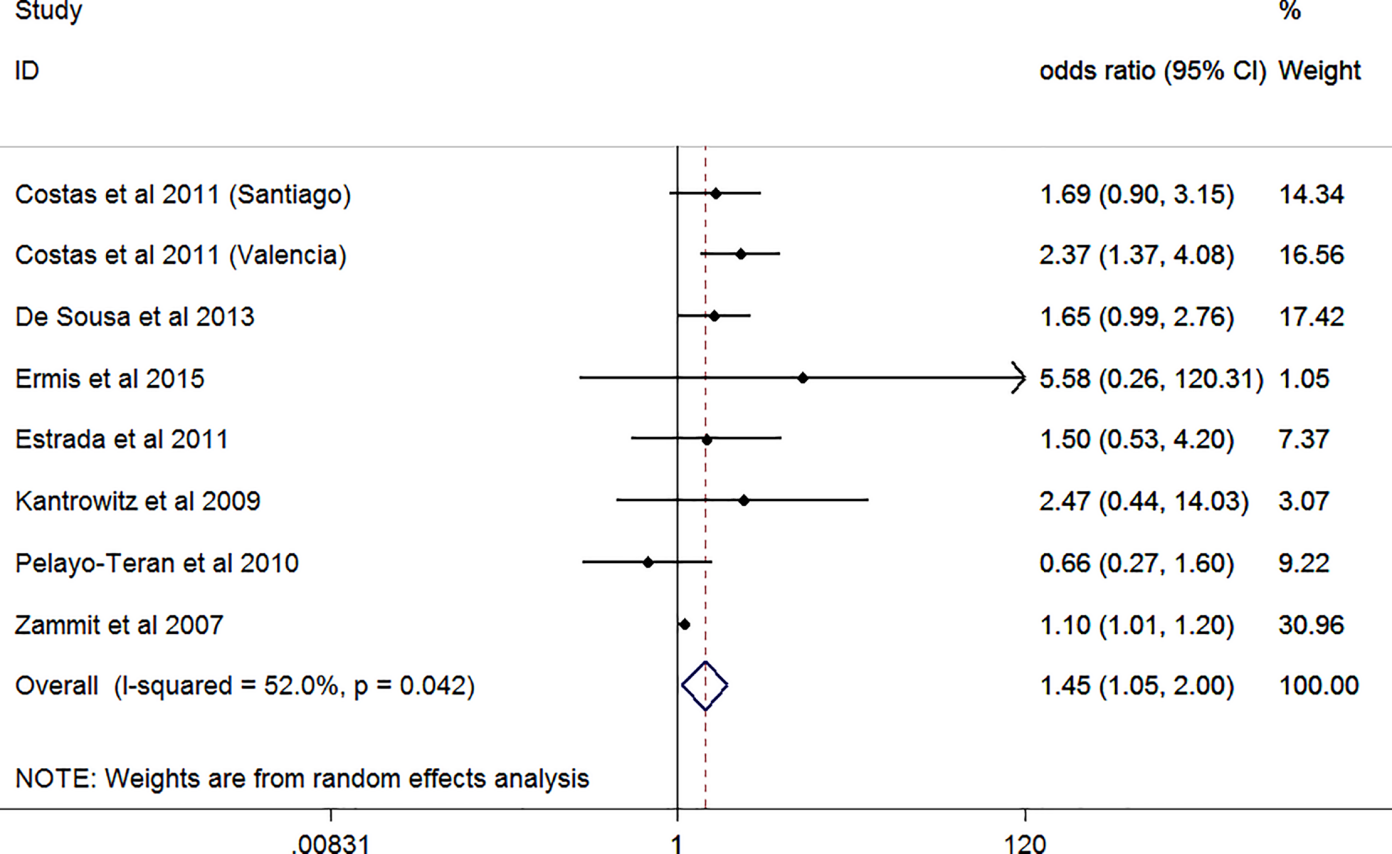
Forest plot case-only studies (figure shows odds ratios; random effects).

In case-only studies, the interaction between cannabis use and COMT^Val158Met^ was statistically significant (OR: 1.45, 95% CI: 1.05–2.0; Fig 2). The I-square of 52% implies heterogeneity. Tau-square was 0.084.When omitting all studies one by one, the OR varied between 1.24 and 1.67 and in 3 instances, the OR was no longer statistically significant (metainf, results available upon request).

**Fig 3:**
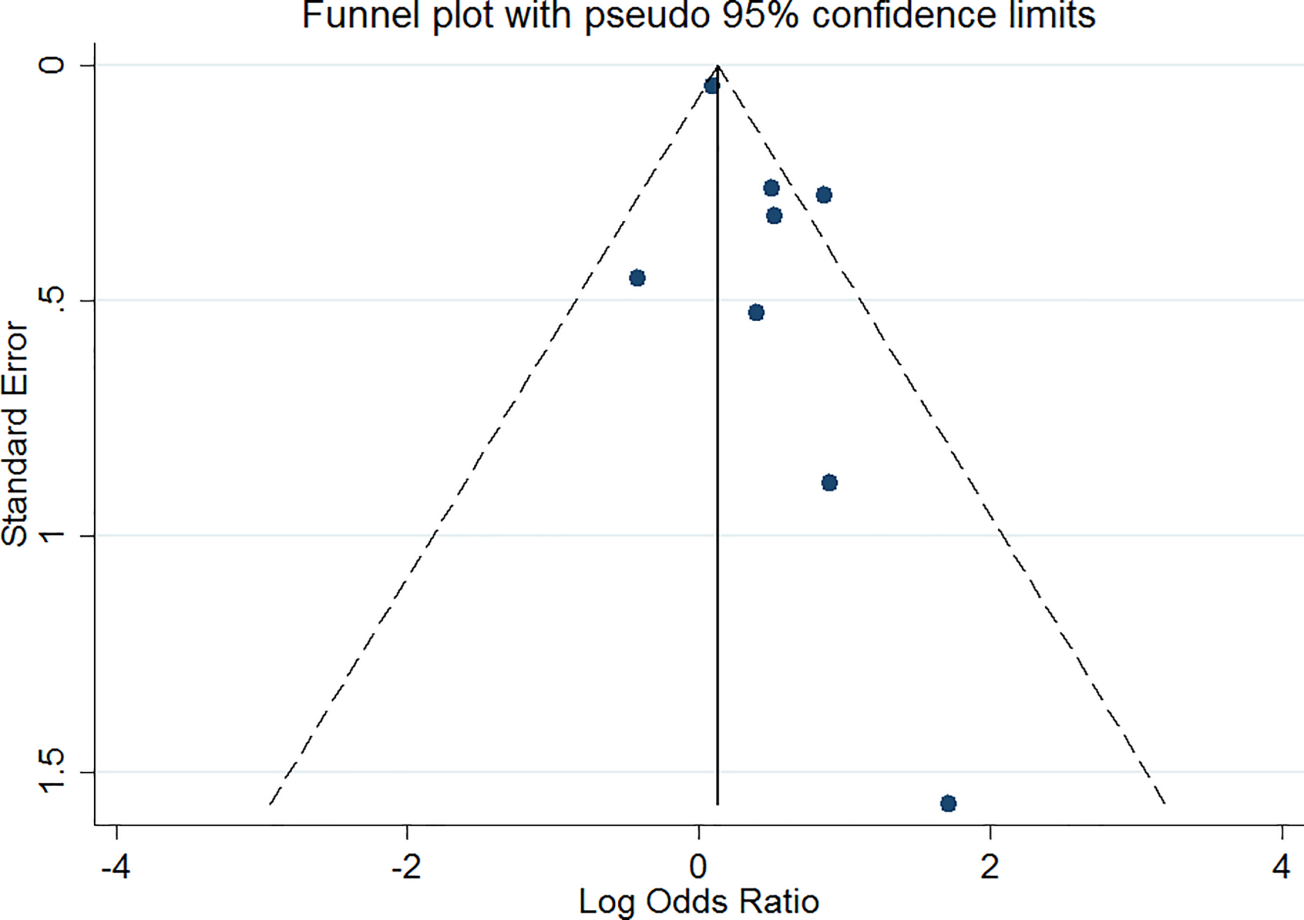
Funnel plot case-only studies.

The Egger test indicated little evidence for publication bias (bias = 1.07, p = 0.086) and the funnel plot in Fig 3 shows some evidence for omitted small negative studies, indicating publication bias. The trim-and-fill method identified 2 missing studies and correcting for this resulted in a small reduction in effect (OR = 1.40, 95% CI 1.04–1.88). Considering that the number of included studies is limited, firm conclusions on publication bias are not possible.

Results from meta-regression showed that neither method of diagnosis (p = 0.73) nor cannabis assessment (p = 0.36), nor high vs low percentages of male sex (p = 0.68) were modifiers.

### Dichotomous outcomes

Table 5 presents study characteristics of the three studies analysing dichotomous outcomes (n = 3433). Validity of these studies is presented in Table 3. Only one study [40] controlled for socioeconomic status. One study [43] assessed current cannabis use rather than cannabis use at adolescence (bias of measurement of interventions: moderate).

**Table 5:** Descriptive statistics dichotomous outcomes.

Article	Sample size Total	Sample size Cases	Assessment cannabis use	Assessment Cases/Controls	Original coding of COMT^Val158Met^	Ethnicity	Sex (% male)
Caspi 2005 [10]	803	21	Cannabis use in adolescence, prospectively at ages 13 and 15 years	DSM-IV schizophreniform	0 (Met/Met) 1 (Val/Met) 2 (Val/Val), reversed in analysis^1^	Caucasian	51.3%
Gutiérrez 2009 [43]^2^	283	91	At least once a week during a minimum of 2 weeks in the preceding month	DSM-IV schizophrenia	0 (Met/Met) 1 (Val/Met) 2 (Val/Val)^2^	Spanish	72.5%
Zammit 2011 [40]	2630	225	At least once	PLIKS-Questionnaires at age 16	0 (Met/Met) 1 (Val/Met) 2 (Val/Val), reversed in analysis^1^	White Ethnicity	No information

^1^ i.e. a minus sign was added to the regression coefficient

^2^ excluded from the meta-analysis because the data needed for extraction were not provided

**Fig 4:**
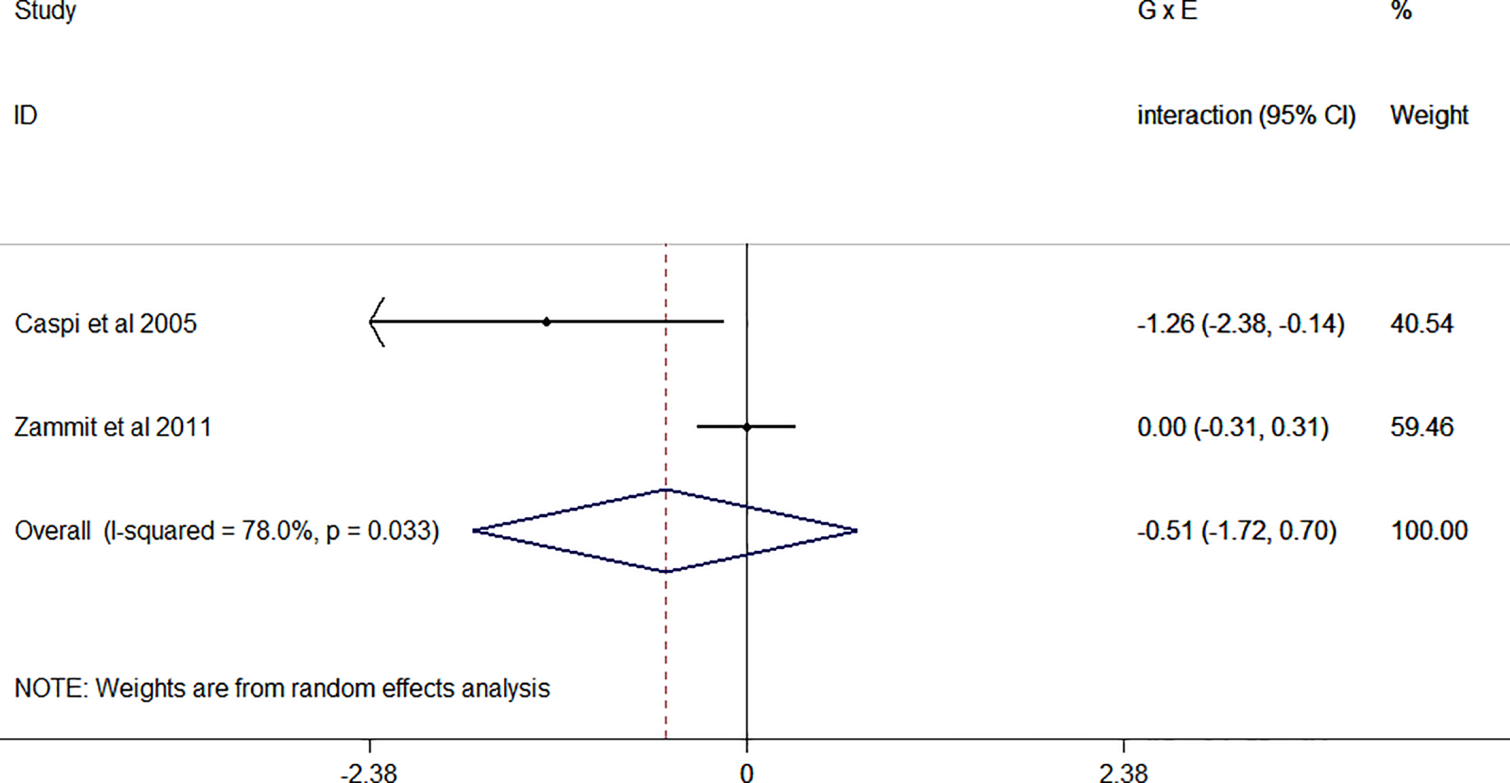
Forest plot dichotomous outcomes (figure shows regression coefficient of the interaction term, random effects).

The forest plot showed no interaction between cannabis use and COMT^Val158Met^ in the dichotomous outcomes (B: -0.51, 95% CI: -1.72–0.70; Fig 4). The study not included in the meta-analysis [43] reported no two-way interaction, but test statistics were not provided. In females, the authors reported the highest psychosis risk in cannabis users who were Val/Val homozygous, but the two-way interaction term was not statistically significant (p = 0.15). This study [43] assessed current cannabis use (at least once a week during a minimum of 2 weeks in the preceding month), which is of limited value as a proxy for cannabis exposure during onset of psychosis.

A sensitivity analysis excluding the study analysing psychotic experiences [40] was not performed because then data of only one study were available for analysis [10]. Results of this study were statistically significant with strongest association between cannabis and psychosis in Val/Val subjects, but the confidence interval was wide.

As the data set included only two studies (two rows in the data), testing modifiers and publication bias was not possible.

### Continuous outcomes

The three studies analysing continuous outcomes included a total of 1823 subjects (Table 6)[35, 41, 42]. One study presented results in a discovery and replication sample; these were treated as two separate studies in the meta-analysis [41]. Two studies made use of the Community Assessment of Psychic Experiences (CAPE) for the assessment of psychotic experiences. In the present analysis, the total CAPE sum score was used. However, in one study [42] the depressive dimension of the CAPE score was not measured and, therefore, the sum score was slightly different [42]. The third study was performed in an ultra-high risk population and used the Comprehensive Assessment of At Risk Mental States (CAARMS; CAARMS positive symptoms sum score selected for the present meta-analysis), a semi-structured interview specifically designed to identify subjects with an elevated risk to develop a first psychosis [35].

**Table 6:** Descriptive statistics continuous outcomes.

Article	Sample size	Assessment cannabis use	Outcome used in the meta-analysis	Original coding of COMT^Val158Met^	Ethnicity	Sex (% male)
Alemany 2014 [42]^2^	419	Cannabis use was assessed with one question regarding the frequency of consumption: ‘never’,‘once’, ‘monthly’, ‘weekly’ or ‘daily’	The Community Assessment of Psychic Experiences (CAPE, total score) was used to assess psychotic experiences (self-report). *General population*.	original paper and extra data: 0 (Val/Val) 1 (Val/Met) 2 (Met/Met)	Caucasian, mostly Spanish	45%
Nieman 2016 [35]^2^	147 (or 123 see ethnicity)	Derived from the Composite International Diagnostic Interview (CIDI)→ at least a period of weekly use.	Comprehensive Assessment of At Risk Mental States; positive symptoms (semi-structured interview). *Ultra-high risk population*.	original paper and extra data: 0 (Met/Met) 1 (Val/Met) 2 (Val/Val) Reversed in analysis^1^	Caucasian, except for 24 non-Caucasians in- and excluded in a sensitivity analysis	48.3%
Vinkers 2013 [41]^2^	Discovery sample: 918 Replication sample: 339	Discovery sample: In the discovery sample, cannabis use was defined as current use more than an equivalent of 3€ euro per week (roughly equivalent to weekly cannabis use) during the last month or longer. Replication sample: In the replication sample, cannabis use was derived from the Composite International Diagnostic Interview (CIDI) with the pattern of cannabis use during the last year as main outcome	The Community Assessment of Psychic Experiences (CAPE, total score) was used to assess psychotic experiences in both samples (self-report). *General population*.	original paper and extra data: 0 (Met/Met) 1 (Val/Met) 2 (Val/Val) Reversed in analysis^1^	All participants were of Dutch ancestry.	Discovery sample: 47%Replication Sample: 43%

^1^ i.e. a minus sign was added to the regression coefficient

^2^ additional data obtained from the authors

Validity of the two general population studies and the ultra-high risk study population (see below) was rather similar (Table 3). One study [41] did not have substantial missing data after genotyping.

**Fig 5:**
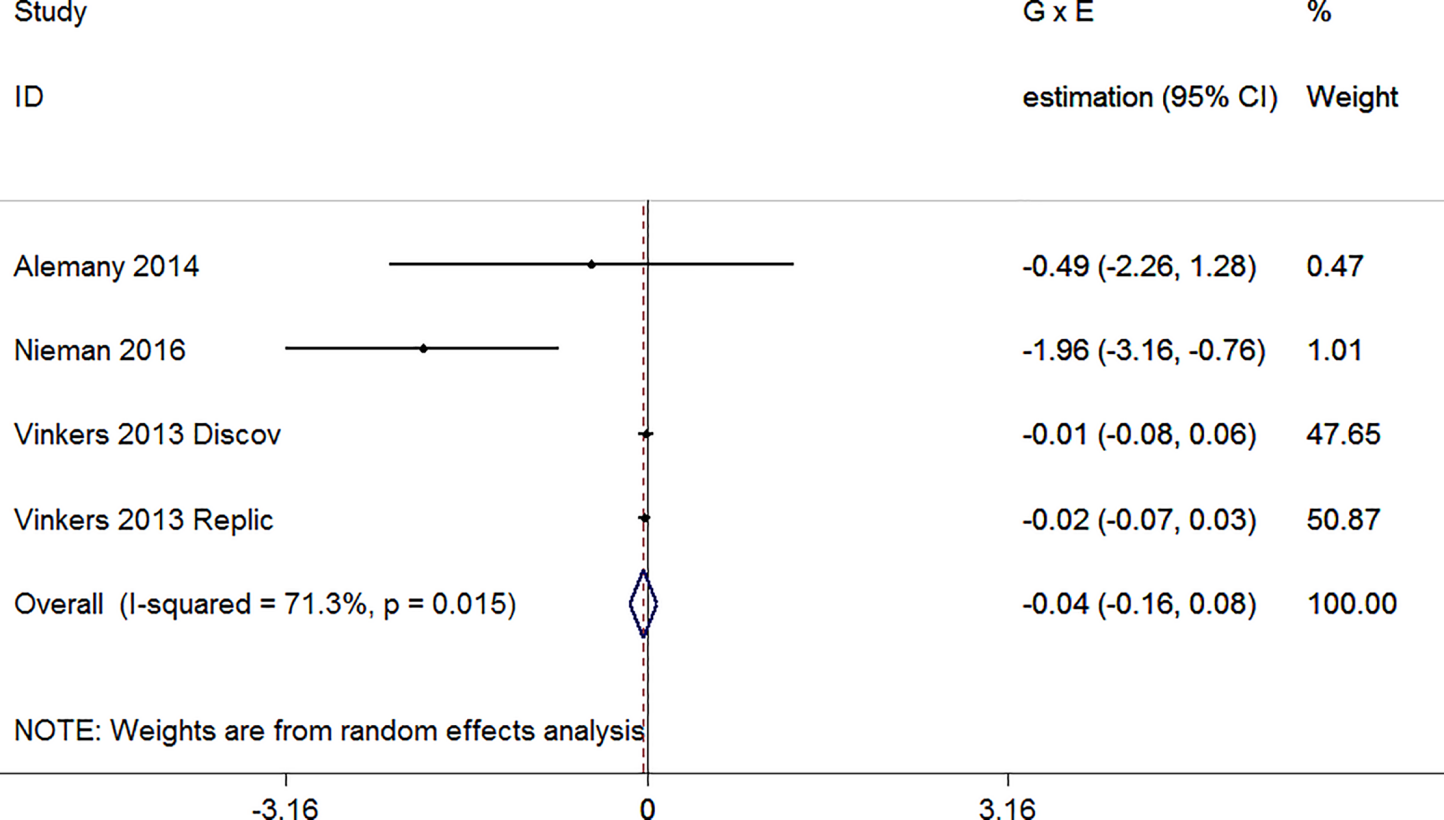
Forest plot continuous outcomes (figure shows regression coefficient of the interaction term, random effects).

In only one of the four samples a significant cannabis X COMT^Val158Met^ interaction was found. Val/Val was associated with a stronger association between cannabis and positive symptoms (negative regression coefficient in the meta-analysis) [35]. Combining all continuous outcomes resulted in an interaction term close to zero (B = -0.04, 95% CI: -0.16–0.08; Fig 5).

When omitting studies (metainf) B was between -0.02 and -0.76, but never statistically significant. Trim and fill did not identify missed studies and Egger’s test for small study effects was not statistically significant (B = -1.86, p = 0.21).

Because only four samples analysing continuous outcomes were included (3 rows because one study included two samples), a meta-regression model including the modifying effect of the variable study (using 1 dummy) was analysed rather than the three modifiers, separately. Although visual inspection of the forest plot showed that the study in the ultra-high risk sample with the significant cannabis X COMT^Val158Met^ interaction was different [35], the variable study (p = 0.30) nor the variable ultra-high risk (p = 0.08) were significant modifiers.

### Sensitivity analysis

A sensitivity analysis excluding the study in the ultra-high risk population showed similar results, but the confidence interval was smaller (B = -0.02, 95% BI -0.06–0.02).

## Discussion

A significant cannabis X COMT^Val158Met^ interaction was found in the case-only studies (OR = 1.43, 95% CI = 1.01; 2.04; Met/Met as the risk genotype), but not in the dichotomous outcomes (B = 0.51; CI -0.70; 1.72) or the continuous outcomes (B = -0.04; CI -0.16; 0.08).

### Methodological issues

The strength of the present study is the power. Using 13 studies in 3 different meta-analyses, we were able to analyse the cannabis X COMT^Val158Met^ interaction including high numbers of individuals (n = 1954, 3433, and 1823, respectively). In order to show interaction effects a very high power is needed. The present null finding is not a consequence of power because the effect size was relevant only in the case-only studies and decreased with the increase of the validity of the included studies in the other two meta-analyses. Thus, it was possible to find an unequivocal null finding. However, the present study has some limitations. First, despite the praised efficiency to find interaction in case-only studies [23], this design has lower methodological validity than the other studies included in the present meta-analysis. Case-only studies have been reported to overestimate the true effects; in particular when the exposure-genotype independence assumption is violated [44]. It is possible that COMT^Val158Met^ causes behavioural changes in cannabis use, causing an imbalance between COMT^Val158Met^ and cannabis that is not a result of the hypothesized interaction. Although the other study types did not provide evidence for gene-environment correlation (see below), the impact of a small violation of the assumption forces us to interpret the case-only results with caution. In addition, this design cannot analyse the main effects of cannabis nor genotype alone, but only the interaction. Conversely, only in the case-only data could we assess the impact of modifiers and publication bias, because this was the largest group of studies.

Second, unfortunately, the number of studies was too small to perform a meta-analysis on cannabis X COMT^Val158Met^ in non-white ethnic groups. Thus, the present null-finding is only valid for the white ethnic group.

Furthermore, the included studies were rather heterogeneous as is often the case in meta-analyses. Not only full-blown psychosis, but also attenuated psychotic symptoms were studied. Although this can be seen as a strength when findings are replicated across various populations, it makes interpretation more difficult when findings are different. However, because only in the design with the lowest quality (case-only) significant cannabis X COMT^Val158Met^ interaction was found, we feel that the present null-finding is valid. The study in the ultra-high risk population also showed this interaction, but this interaction was reversed.

Finally, most included studies were cross-sectional. Case-control studies when analysing DSM-diagnoses as well as cohort studies when analysing continuous outcomes are scarce. All studies except one analysed cannabis use during adolescence, introducing a longitudinal element in the cross-sectional data. However, when cross-sectional results do not show an interaction, longitudinal data are also unlikely to show interaction.

### Flip-flop

There is a debate on the existence of flip-flop and how to analyse data when flip-flop is observed [32]. Flip-flop describes the phenomenon that in different populations a different allele of the single nucleotide polymorphism (SNP) is associated with the disease. This is the case when not the studied SNP, but a SNP close to that location mediates the risk for a disease. In other words, due to rare cross-over events during conception the disease can be associated with either the one or the other allele of the SNP in different populations. In the present paper, we conservatively assumed no flip-flop [32, 45].

While the pooled effect in the case-only studies identified Met/Met as the risk genotype, in one case-only study Val/Val was the risk genotype [31] n.s.). In addition, in two of the four continuous outcome samples ([42] n.s., [35]) and two dichotomous outcome studies ([10] n.s. [43] females excluded) Val/Val was identified as the risk, while the other results were null findings.

When results of all these studies were reversed as suggested by the flip-flop theory, the OR for interaction in case-only studies was 1.55 (1.14–2.10), while the regression coefficients for interaction were 0.51 (-0.70–1.72) and 0.01 (-0.12–0.13) in studies with dichotomous and continuous outcomes, respectively. Thus, when assuming flip-flop results are rather similar to the original results.

### Ultra-high risk

Given that ultra-high risk samples majorly consist of individuals with ‘attenuated psychotic symptoms’, that conceptually and psychometrically resemble the concept of ‘psychotic experiences’ in general population studies [46, 47], the ultra-high risk sample and the general population samples were combined in the same meta-analysis. Although a sensitivity analysis excluding the ultra-high risk paper yielded very similar results (B = -0.02; CI -0.06; 0.02), results in the ultra-high risk group were rather different (B = -1.96; CI -3.16; -0.76; meta-regression coefficient of the difference between general population and ultra-high risk = n.s.). One explanation is that the outcome parameter in the ultra-high risk study was total score on the positive items of the CAARMS whereas in the other continuous outcome studies, CAPE total score (that also includes negative and depressive symptom items) was used. However, the three CAPE scores in practice are very strongly correlated with each other (in the order of 0.7–0.8)[48], suggesting they tap into the same underlying dimension and making it unlikely that use of total CAPE score explains differential findings with other outcomes. In addition, the ultra-high risk study interviewed subjects using the CAARMS, while the general population studies used the CAPE self-report.

### Discussion of main findings

There may be two reasons why results of the case-only meta-analysis are different from the other results. First, the dichotomous and continuous outcomes meta-analyses analysed studies with a superior study design compared to the case-only studies. Second, case-only designs may overestimate the true effects [44] as was discussed above. Despite the large numbers of subjects included in the case-only meta-analysis, the lower limit of the confidence interval was close to no effect (OR = 1). Correction for publication bias in the case-only studies decreased the effect, further supporting the null-finding in the other study types. Moreover, the low replication rate of gene–environment interactions in general is evidence for publication bias in this research area [49], but despite this the overall result points at a null finding.

By contrast, some results of the dichotomous and continuous outcome results that were not included in the data set for the meta-analysis did suggest interaction between cannabis and COMT^Val158Met^. For example, when continuous outcomes were standardised, the cannabis X COMT^Val158Met^ interaction in the sum score of positive symptoms was more than 4 times as large as in the total sum score and statistically significant (30). Second, two continuous outcome studies included child maltreatment as a third interaction variable in a three-way interaction. When unravelling the regression models including the two-way interaction and the three-way interaction, the two-way interaction in the Vinkers discovery sample was 0.39 (p = 0.006) and 0.1 in non-abused and abused children respectively, while the two-way interaction in the Alemany sample was -0.452 (n.s.) and -0.755 (significance unknown) in non-maltreated and maltreated children [42]. In the Vinkers replication sample both two-way and three-way interaction terms were close to zero [41].

Third, Caspi analysed various dichotomous outcomes [10]. Diagnosis of schizophreniform disorder (B = 1.26, p = 0.025) was included in the present meta-analysis. The cannabis X COMT^Val158Met^ interaction was not statistically significant when analysing self-report of psychotic symptoms and evidence of hallucinatory experiences (B = 0.88, p = 0.49 and B = 0.73, p = 0.21 respectively).There was no interaction when studying delusional beliefs and informant reports of psychotic symptoms.

Additionally, Pelayo—Teran [31] showed a related interaction. Cannabis prevented the protective effects of the met variant of COMT^Val158Met^. Age of onset is earlier in both patients with the val variant and in patients with the met variant using cannabis. Finally, one study reported a cannabis X COMT^Val158Met^ interaction in females only, but this interaction was not statistically significant (31).

Similar to the case-only meta-analysis, most above-mentioned results do suggest evidence for gene-environment interaction. However, results are isolated and inconsistent. Moreover, if similar analyses in subgroups of other studies resulted in a null finding, publication bias is likely [49]. Thus, there is little evidence for an interaction between cannabis and COMT^Val158Met^ in the psychosis phenotype.

### Support in the literature

Although main effects of COMT^Val158Met^ have been reported earlier [50], recent work showed that the association between COMT^Val158Met^ and schizophrenia may be inconsistent [51, 52]. However, the presence of a main effect is not a condition to warrant studying interaction. The number of individual studies on the cannabis X COMT^Val158Met^ interaction as well as the ongoing debate warranted a meta-analysis. In addition, while GWAS showed small effects of SNPs, both individual and cumulative, the importance of gene-gene and gene-environment interactions has been emphasized [53]. For example, the COMT^Val158Met^ val/val variant may increase vulnerability to cannabis [53] or cannabis may be associated with epigenetic modulation of the COMT^Val158Met^ gene [54].

Besides the epigenetic modulation various other mechanisms have been suggested, but there is no agreement. Two recent publications provide a summary of the current state of knowledge [52, 55]. In short, COMT^Val158Met^ is involved in dopamine regulation in the brain. One of the cannaboid receptors (CB1) reacts not only to endogenous cannabinoids, but also to THC, thus potentially establishing a link between THC and dopamine levels. The COMT^Val158Met^ val/val variant may significantly worsen the effects of THC on for example cognition through its impact on dopaminergic neurotransmission [52].

An interaction between cannabis and COMT^Val158Met^ is plausible when examining experiments in humans and laboratory animals. First, several rodent studies have addressed this subject. O’Tuathaigh and colleagues [56] showed that THC administration in COMT^Val158Met^ knockout mice was more strongly associated with indicators for psychosis related phenotypes in humans than in wild type mice. Furthermore, Batalla and colleagues [57] studied neuroanatomical changes after cannabis use in a neuroimaging study. They showed that these changes were modulated by the COMT^Val158Met^ gene [57]. A laboratory study in humans indicated that the behavioural response to THC is moderated by COMT^Val158Met^ [58]. On the other hand, COMT^Val158Met^ did not impact on the association between direct THC administration and the CAPE total score [59]. The above-mentioned results on mechanisms are scarce and the research on the mechanisms why cannabis causes psychosis is still in its infancy [2].When mechanisms of the main effect are unclear, mechanisms of the gene–environment interaction are even more difficult to study. The lack of undisputed mechanisms further supports the null finding.

As stated in the introduction, gene-environment interaction in psychosis and psychotic disorders does not only include cannabis X COMT^Val158Met^. Besides the association between cannabis and psychosis, multiple other environmental factors may play a role. Examples include childhood trauma [60] and urbanicity [61]. Initially, researchers tried to identify a single or a limited number of locations on the DNA as risk loci for various diseases (association studies), including the psychotic phenotype. The COMT^Val158Met^ gene was among the few that were widely studied and, therefore, this was the only genetic variation that could be included in the current meta-analysis. Currently, a large number of genetic loci with small effect sizes are associated with the psychotic phenotype. For this reason, the polygenic risk sore for schizophrenia was constructed [62]. Instead of analysing each locus individually, currently the focus is on the polygenic risk score, to avoid multiple testing and to increase power. Thus, the cannabis X COMT^Val158Met^ interaction is only one of multiple gene—environment interactions that are plausible to co-exist. Although including the schizophrenia polygenic risk score in gene-environment studies is advocated [63], these types of studies are scarce. A recent study reported that the polygenic risk score defining genetic risk for schizophrenia was a modifier in the association between cannabis and brain maturation in males [64]. In contrast, a pilot study did not show evidence for interaction between polygenic risk score and childhood trauma in psychosis [65]. Thus, although there is no evidence for an interaction between cannabis and COMT^Val158Met^, interaction with other genetic risk factors has not been studied. More research using the polygenic risk score of schizophrenia or other more sophisticated genetic assessments are needed before this can be analysed in a meta-analysis.

### Gene environment correlation

It has been argued that gene-environment interaction can only be studied when there is no gene-environment correlation [1]. Not all studies included in the meta-analyses reported on gene-environment correlation. In the case-only studies, this analysis is impossible because it overlaps with the assumption used for this type of study. In the other six studies, there was no gene-environment correlation in 2 studies [10, 35, 40, 42]. The other authors did not report gene-environment correlation [41, 43]. Because more than half of the samples did not have gene-environment correlation in their data, we feel that gene-environment correlation in this meta-analysis is unlikely.

## Conclusion and suggestions for further research

In conclusion, the present meta-analysis did not show evidence for an interaction between cannabis and COMT^Val158Met^ when studying psychotic symptoms or psychotic disorder. For future studies, multiple other factors should be taken into account. The analysis of gene-environment interplay may provide useful information about the development and treatment of psychotic disorders.

In the future, researchers should invest in higher quality research designs rather than performing another case-only study. In addition, as has been advocated previously [2, 49], the present results show once again that replication is always highly needed; a single positive study result can hardly be seen as evidence. When replications are performed, we would urge researchers to use a standard set of instruments. That would make pooling in a meta-analysis easier. Currently, for the diagnosis of psychotic disorder, DSM IV or 5 or the Structured Clinical Interview (SCID) for DSM-IV are exchangeable. When assessing psychotic symptoms or attenuated psychotic symptoms in the general population and in ultra-high risk populations the preferred instruments are the CAPE and the CAARMS, respectively. Therefore, we would advise to include those instruments in future research.

## Supporting information

S1 File Prisma 2009 checklist. (DOC)

S2 File Excel data case-only studies. (XLSX)

S3 File Excel data dichotomous outcome studies. (XLSX)

S4 File Excel data continuous outcome studies. (XLSX)
