# Supplementary material for: The interaction between cannabis use and the Val158Met polymorphism of the COMT gene in psychosis: A transdiagnostic meta – analysis
Source: PLoS One. 2018 Feb 14;13(2):e0192658. doi: 10.1371/journal.pone.0192658 (PMC5812637; doi:10.1371/journal.pone.0192658)
Supplement: S1 File — (DOC) [file pone.0192658.s001.doc]

| **Section/topic** | **#** | **Checklist item** | **Reported on page #** |
| --- | --- | --- | --- |
| **TITLE** | | |  |
| Title | 1 | Identify the report as a systematic review, meta-analysis, or both.  The interaction between cannabis use and the Val158Met polymorphism of the COMT gene in psychosis: a transdiagnostic meta – analysis | 1 |
| **ABSTRACT** | | |  |
| Structured summary | 2 | Provide a structured summary including, as applicable: background; objectives; data sources; study eligibility criteria, participants, and interventions; study appraisal and synthesis methods; results; limitations; conclusions and implications of key findings; systematic review registration number  **Background** Neither environmental nor genetic factors are sufficient to predict the transdiagnostic expression of psychosis. Therefore, analysis of gene-environment interactions may be productive.  **Objective.** A meta-analysis was performed using papers investigating the interaction between cannabis use and catechol-O-methyl transferase (COMT) polymorphism Val158Met (COMT^Val158Met^).  **Data sources.** Pubmed, Embase, PsychInfo  **Study eligibility criteria.** All observational studies assessing the interaction between COMT^Val158Met^ and cannabis with any psychosis or psychotic symptoms measure as an outcome.  **Study appraisal and synthesis methods.** A meta-analysis was performed using the Meta-analysis of Observational Studies in Epidemiology guidelines and forest plots were generated. Thirteen articles met the selection criteria: 7 clinical studies using a case-only design, 3 clinical studies with a dichotomous outcome, and 3 studies analysing a continuous outcome of psychotic symptoms below the threshold of psychotic disorder. The three study types were analysed separately. Validity of the included studies was assessed using "A Cochrane Risk of Bias Assessment Tool: for Non-Randomized Studies of Interventions".  **Results** For case-only studies, a significant interaction was found between cannabis use and COMT^Val158Met^, with an OR of 1.45 (95% Confidence Interval = 1.05-2.00; for Met/Met as the risk genotype). However, there was no evidence for interaction in either the studies including dichotomous outcomes (B=-0.51, 95% Confidence Interval -1.72, 0.70) or the studies including continuous outcomes (B=-0.04 95% Confidence Interval -0.16 – 0.08).  **Limitation.** A substantial part of the included studies used the case-only design, which has lower validity and tends to overestimate true effects.  **Conclusion** Considering that the interaction term was only statistically significant in the case-only studies, but not in studies using other clinical or non-clinical psychosis outcomes, there is no convincing evidence for interaction between cannabis use and COMT^Val158Met^. | 2 |
| **INTRODUCTION** | | |  |
| Rationale | 3 | Describe the rationale for the review in the context of what is already known.  Previously various case-only studies evidenced interaction between COMT^Val158Met^ and cannabis when studying psychosis. However, other studies had conflicting results. With this meta-analysis we aimed to get a final true answer on this research question. | 6/7 |
| Objectives | 4 | Provide an explicit statement of questions being addressed with reference to participants, interventions, comparisons, outcomes, and study design (PICOS).  Participants – *subjects*: patient populations, general populations and ultra high risk populations.  Interventions vs comparisons – *the independent variable*: the interaction between COMT^Val158Met^ and cannabis.  Outcomes: the diagnosis of psychosis as well as continuous variables assessing psychotic symptoms in the general population, expressed in odds ratio's (case-only studies) or regression coefficients (other study types).  Study designs: all (case-only, case-control (dichotomous outcome), cross-sectional, cohort) | P 6  I, C 6  O 7 9/10  S 7, 9 |
| **METHODS** | | |  |
| Protocol and registration | 5 | Indicate if a review protocol exists, if and where it can be accessed (e.g., Web address), and, if available, provide registration information including registration number.  A review protocol doesn't exist. We followed the MOOSE criteria | 7 |
| Eligibility criteria | 6 | Specify study characteristics (e.g., PICOS, length of follow-up) and report characteristics (e.g., years considered, language, publication status) used as criteria for eligibility, giving rationale.  PICOS see question #4. We had no criteria on study duration. All included studies were cross-sectional or case-control. | 6, 7, 9, 10 |
| Information sources | 7 | Describe all information sources (e.g., databases with dates of coverage, contact with study authors to identify additional studies) in the search and date last searched.  We searched Pubmed, Embase, PsychInfo, July 18^th^ 2017.  All publication years in the databases and articles in English, German and Dutch were included in the search. In addition, no exclusion was conducted based on the type of study.  All authors of studies that should be included, but didn't provide the information we needed were e-mailed. When they didn't respond up to two reminders were sent.  We didn't contact authors to identify additional studies because we aimed to include published studies only (peer-reviewed). | 7 |
| Search | 8 | Present full electronic search strategy for at least one database, including any limits used, such that it could be repeated. | 8 |
| Study selection | 9 | State the process for selecting studies (i.e., screening, eligibility, included in systematic review, and, if applicable, included in the meta-analysis).  See flow-diagram | fig. 1 |
| Data collection process | 10 | Describe method of data extraction from reports (e.g., piloted forms, independently, in duplicate) and any processes for obtaining and confirming data from investigators.  Only published articles were included. Both study selection and data extraction were performed by two authors independently. Authors were asked for more information if they studied the research question, but didn’t provide the data we needed. | 9 |
| Data items | 11 | List and define all variables for which data were sought (e.g., PICOS, funding sources) and any assumptions and simplifications made.  Regression coefficient of interaction term cannabis X COMT^Val158Met^ or a proxy of this in case-only studies (OR in 2x2 table cannabis X COMT^Val158Met^ in cases. Assumption: When a case-only study design to provide evidence for gene-environment interaction is used, the main assumption is that the prevalences of the environmental factor and the genotype are independent of each other in the population (no gene-environment correlation). | 9/10 (assumption 5) |
| Risk of bias in individual studies | 12 | Describe methods used for assessing risk of bias of individual studies (including specification of whether this was done at the study or outcome level), and how this information is to be used in any data synthesis.  When sufficient data was available (case-only studies), modifiers were analysed using the *metareg* command (diagnosis using DSM IV vs revised DSM IV, cannabis use never versus ever cf less stringent criteria, >70% male sex vs <70% male sex).  The *metainf* command was used to check single study effects.  Publication bias was tested using the *metafunnel* command to generate a funnel plot, the *metatrim* command to identify the possibility of unpublished negative findings and to control for that (trim and fill) [32] as well as the *metabias* command to obtain Egger's test for small study effects. | 10 |
| Summary measures | 13 | State the principal summary measures (e.g., risk ratio, difference in means).  Case-only studies: odds ratios  Other studies: regression coefficient of the interaction effect | 9/10 |
| Synthesis of results | 14 | Describe the methods of handling data and combining results of studies, if done, including measures of consistency (e.g., I^2^) for each meta-analysis.  The *metan* command provided the between study variance (tau-square) and the Higgins I-square, which is a measure of heterogeneity. We performed 3 meta-analyses using the metan command because we couldn’t combine the three types of studies into one meta-analysis. | 10 |

Page 1 of 2

| **Section/topic** | **#** | **Checklist item** | **Reported on page #** |
| --- | --- | --- | --- |
| Risk of bias across studies | 15 | Specify any assessment of risk of bias that may affect the cumulative evidence (e.g., publication bias, selective reporting within studies).  There might be some publication bias in the case-only studies. Correction for publication bias in the case-only studies decreased the effect, further supporting the null-finding in the other study types.  In addition, we do expect bias (towards a larger effect) in the case-only studies. So taking into account both types of bias our conclusion is that there is no evidence for interaction cannabis X COMT^Val158Met^; a null finding. | 24 |
| Additional analyses | 16 | Describe methods of additional analyses (e.g., sensitivity or subgroup analyses, meta-regression), if done, indicating which were pre-specified.  We analysed the three types of studies, separately, because they couldn't be combined. When analysing the continuous outcomes we excluded the ultra high risk sample in a sensitivity analysis, because this population is rather different from the included general population samples.  When sufficient data was available (case-only studies), modifiers were analysed using the *metareg* command (diagnosis using DSM IV vs revised DSM IV, cannabis use never versus ever cf less stringent criteria, >70% male sex vs <70% male sex). | 3 types of studies separately: 7  sensitivity analysis: 11  meta-regression 10 |
| **RESULTS** | | |  |
| Study selection | 17 | Give numbers of studies screened, assessed for eligibility, and included in the review, with reasons for exclusions at each stage, ideally with a flow diagram.  See flow diagram | Fig 1 |
| Study characteristics | 18 | For each study, present characteristics for which data were extracted (e.g., study size, PICOS, follow-up period) and provide the citations.  Study characteristics in table 3, 5 and 6 in the article.  Citation:  10. Caspi A, Moffitt TE, Cannon M, McClay J, Murray R, Harrington H, et al. Moderation of the effect of adolescent-onset cannabis use on adult psychosis by a functional polymorphism in the catechol-O-methyltransferase gene: longitudinal evidence of a gene X environment interaction. Biological psychiatry. 2005;57(10):1117-27.  19. Estrada G, Fatjó-Vilas M, Munoz M, Pulido G, Minano M, Toledo E, et al. Cannabis use and age at onset of psychosis: further evidence of interaction with COMT Val158Met polymorphism. Acta psychiatrica Scandinavica. 2011;123(6):485-92.  21. Costas J, Sanjuán J, Ramos-Ríos R, Paz E, Agra S, Tolosa A, et al. Interaction between COMT haplotypes and cannabis in schizophrenia: a case-only study in two samples from Spain. Schizophrenia research. 2011;127(1):22-7.  26. Kantrowitz JT, Nolan KA, Sen S, Simen AA, Lachman HM, Bowers Jr MB. Adolescent cannabis use, psychosis and catechol-O-methyltransferase genotype in African Americans and Caucasians. Psychiatric quarterly. 2009;80(4):213-8.  29. Pelayo-Terán JM, Pérez-Iglesias R, Mata I, Carrasco-Marín E, Vázquez-Barquero JL, Crespo-Facorro B. Catechol-O-Methyltransferase (COMT) Val158Met variations and cannabis use in first-episode non-affective psychosis: clinical-onset implications. Psychiatry research. 2010;179(3):291-6.  33. Nieman DH, Dragt S, van Duin ED, Denneman N, Overbeek JM, de Haan L, et al. COMT Val(158)Met genotype and cannabis use in people with an At Risk Mental State for psychosis: Exploring Gene x Environment interactions. Schizophr Res. 2016;174(1-3):24-8. doi: 10.1016/j.schres.2016.03.015. PubMed PMID: 27052366.  34. Zammit S, Spurlock G, Williams H, Norton N, Williams N, O’DONOVAN MC, et al. Genotype effects of CHRNA7, CNR1 and COMT in schizophrenia: interactions with tobacco and cannabis use. The British Journal of Psychiatry. 2007;191(5):402-7.  35. De Sousa KR, Tiwari AK, Giuffra DE, Mackenzie B, Zai CC, Kennedy JL. Age at onset of schizophrenia: cannabis, COMT gene, and their interactions. Schizophr Res. 2013;151(1-3):289-90. doi: 10.1016/j.schres.2013.10.037. PubMed PMID: 24268936.  36. Ermis A, Erkiran M, Dasdemir S, Turkcan AS, Ceylan ME, Bireller ES, et al. The relationship between catechol-O-methyltransferase gene Val158Met (COMT) polymorphism and premorbid cannabis use in Turkish male patients with schizophrenia. In Vivo. 2015;29(1):129-32. PubMed PMID: 25600541.  37. Zammit S, Owen MJ, Evans J, Heron J, Lewis G. Cannabis, COMT and psychotic experiences. The British Journal of Psychiatry. 2011;199(5):380-5.  38. Vinkers CH, Van Gastel WA, Schubart CD, Van Eijk KR, Luykx JJ, Van Winkel R, et al. The effect of childhood maltreatment and cannabis use on adult psychotic symptoms is modified by the COMT Val 158 Met polymorphism. Schizophrenia research. 2013;150(1):303-11.  39. Alemany S, Arias B, Fatjó-Vilas M, Villa H, Moya J, Ibanez M, et al. Psychosis‐inducing effects of cannabis are related to both childhood abuse and COMT genotypes. Acta psychiatrica Scandinavica. 2014;129(1):54-62.  40. Gutierrez B, Rivera M, Obel L, McKenney K, Martinez-Leal R, Molina E, et al. Variability in the COMT gene and modification of the risk of schizophrenia conferred by cannabis consumption. Rev Psiquiatr Salud Ment. 2009;2(2):89-94. doi: 10.1016/S1888-9891(09)72250-5. PubMed PMID: 23034243. | 13, 18, 19,  31-33 |
| Risk of bias within studies | 19 | Present data on risk of bias of each study and, if available, any outcome level assessment (see item 12).  A summary of the validity of the studies is provided in table 4 in the article. | 15 |
| Results of individual studies | 20 | For all outcomes considered (benefits or harms), present, for each study: (a) simple summary data for each intervention group (b) effect estimates and confidence intervals, ideally with a forest plot.  See figure 2, 4 and 5 in the article | Fig 2, Fig 4, Fig 5. |
| Synthesis of results | 21 | Present results of each meta-analysis done, including confidence intervals and measures of consistency.  See figure 2, 4 and 5 in the article | Fig 2, Fig 4, Fig 5. |
| Risk of bias across studies | 22 | Present results of any assessment of risk of bias across studies (see Item 15).  See figure 3 in the article.  The Egger test indicated little evidence for publication bias (bias=1.07, p=0.086) and the funnel plot in Fig 3 shows some evidence for omitted small negative studies, indicating publication bias. The trim-and-fill method identified 2 missing studies and correcting for this resulted in a small reduction in effect (OR=1.40, 95% CI 1.04-1.88). Considering that the number of included studies is limited, firm conclusions on publication bias are not possible.  Results from meta-regression showed that neither method of diagnosis (p=0.73) nor cannabis assessment (p=0.36), nor high vs low percentages of male sex (p=0.68) were modifiers.  Similar paragraphs for the dichotomous outcomes and the continuous outcomes. | Fig 3 and page 16, 20, 20/21 |
| Additional analysis | 23 | Give results of additional analyses, if done (e.g., sensitivity or subgroup analyses, meta-regression [see Item 16]).  See answer #22  A sensitivity analysis excluding the study in the ultra-high risk population showed similar results, but the confidence interval was smaller (B=-0.02, 95% BI -0.06 - 0.02). | 21 |
| **DISCUSSION** | | |  |
| Summary of evidence | 24 | Summarize the main findings including the strength of evidence for each main outcome; consider their relevance to key groups (e.g., healthcare providers, users, and policy makers).  In conclusion, the present meta-analysis did not show evidence for an interaction between cannabis and COMT^Val158Met^ when studying psychotic symptoms or psychotic disorder.  Relevance: Our final answer using all available evidence is a null finding. This contradicts earlier literature and fragmented evidence for this interaction. The earlier belief that potential future cannabis users should test their COMT status before deciding to use cannabis or not is refuted. On the other hand, both cannabis and COMT^Val158Met^ are risk factor for psychotic symptoms and full blown psychotic disorder and policy makers and healthcare providers should still discourage cannabis use and testing subjects' COMT status is still interesting because of the main effect. | 27 |
| Limitations | 25 | Discuss limitations at study and outcome level (e.g., risk of bias), and at review-level (e.g., incomplete retrieval of identified research, reporting bias).  A substantial part of the included studies used the case-only design, which has lower validity and tends to overestimate true effects. | 21/22 (and page 2) |
| Conclusions | 26 | Provide a general interpretation of the results in the context of other evidence, and implications for future research.  For future studies, multiple other factors should be taken into account. The analysis of gene-environment interplay may provide useful information about the development and treatment of psychotic disorders. | 27 |
| **FUNDING** | | |  |
| Funding | 27 | Describe sources of funding for the systematic review and other support (e.g., supply of data); role of funders for the systematic review.  No funding | - |

*From:*  Moher D, Liberati A, Tetzlaff J, Altman DG, The PRISMA Group (2009). Preferred Reporting Items for Systematic Reviews and Meta-Analyses: The PRISMA Statement. PLoS Med 6(7): e1000097. doi:10.1371/journal.pmed1000097

For more information, visit: **www.prisma-statement.org**.

Page 2 of 2
